# Identification of gene fusion transcripts by transcriptome sequencing in *BRCA1*-mutated breast cancers and cell lines

**DOI:** 10.1186/1755-8794-4-75

**Published:** 2011-10-27

**Authors:** Kevin CH Ha, Emilie Lalonde, Lili Li, Luca Cavallone, Rachael Natrajan, Maryou B Lambros, Costas Mitsopoulos, Jarle Hakas, Iwanka Kozarewa, Kerry Fenwick, Chris J Lord, Alan Ashworth, Anne Vincent-Salomon, Mark Basik, Jorge S Reis-Filho, Jacek Majewski, William D Foulkes

**Affiliations:** 1 Department of Human Genetics, McGill University, Room N5-13, Stewart Biology Building, 1205 Dr. Penfield Ave, Montreal, Quebec, H3A 1B1, Canada; 2 McGill University and Genome Quebec Innovation Centre, 740 Dr. Penfield Ave, Montreal, Quebec, H3A 1A4, Canada; 3 Program in Cancer Genetics, McGill University, 546 Pine Ave, Montreal, Quebec, H2W 1S6, Canada; 4 Segal Cancer Centre, Lady Davis Institute, Jewish General Hospital, 3755 Côte-Ste-Catherine Road, Montreal, Quebec, H3T 1E2, Canada; 5 The Breakthrough Breast Cancer Research Centre, The Institute of Cancer Research, 237 Fulham Road, London, SW3 6JB, UK; 6 Institut Curie, 26 Rue d'Ulm, 75248, Paris, France; 7 Department of Oncology, Lady Davis Institute, Jewish General Hospital, McGill University, 3755 Côte-Ste-Catherine Road, Montreal, H3T 1E2, Canada; 8 Department of Surgery, Lady Davis Institute, Jewish General Hospital, McGill University, 3755 Côte-Ste-Catherine Road, Montreal, Quebec, H3T 1E2, Canada

## Abstract

**Background:**

Gene fusions arising from chromosomal translocations have been implicated in cancer. However, the role of gene fusions in *BRCA1*-related breast cancers is not well understood. Mutations in *BRCA1 *are associated with an increased risk for breast cancer (up to 80% lifetime risk) and ovarian cancer (up to 50%). We sought to identify putative gene fusions in the transcriptomes of these cancers using high-throughput RNA sequencing (RNA-Seq).

**Methods:**

We used Illumina sequencing technology to sequence the transcriptomes of five *BRCA1*-mutated breast cancer cell lines, three *BRCA1*-mutated primary tumors, two secretory breast cancer primary tumors and one non-tumorigenic breast epithelial cell line. Using a bioinformatics approach, our initial attempt at discovering putative gene fusions relied on analyzing single-end reads and identifying reads that aligned across exons of two different genes. Subsequently, latter samples were sequenced with paired-end reads and at longer cycles (producing longer reads). We then refined our approach by identifying misaligned paired reads, which may flank a putative gene fusion junction.

**Results:**

As a proof of concept, we were able to identify two previously characterized gene fusions in our samples using both single-end and paired-end approaches. In addition, we identified three novel in-frame fusions, but none were recurrent. Two of the candidates, *WWC1-ADRBK2 *in HCC3153 cell line and *ADNP-C20orf132 *in a primary tumor, were confirmed by Sanger sequencing and RT-PCR. RNA-Seq expression profiling of these two fusions showed a distinct overexpression of the 3' partner genes, suggesting that its expression may be under the control of the 5' partner gene's regulatory elements.

**Conclusions:**

In this study, we used both single-end and paired-end sequencing strategies to discover gene fusions in breast cancer transcriptomes with *BRCA1 *mutations. We found that the use of paired-end reads is an effective tool for transcriptome profiling of gene fusions. Our findings suggest that while gene fusions are present in some *BRCA1*-mutated breast cancers, they are infrequent and not recurrent. However, private fusions may still be valuable as potential patient-specific biomarkers for diagnosis and treatment.

## Background

Gene fusions are the result of aberrant chromosomal translocations that joins together the exons of two unrelated genes, producing a chimeric mRNA transcript and protein. Many gene fusions that contribute to oncogenesis have been described in literature [1], such as the well-documented *BCR-ABL *in chronic myelogenous leukemia [2]. Recently, there has been greater interest in utilizing massively parallel RNA sequencing (RNA-Seq) data to identify gene fusions [3-5]. RNA-Seq has emerged as a powerful tool to profile the entire transcriptome at a level of detail unattainable by microarrays [6,7]. Here, we sought to identify putative gene fusion mRNA transcripts in *BRCA1*-mutated breast cancers.

Mutations in *BRCA1 *(and *BRCA2*) confer a high risk for breast cancer, with a lifetime risk of up to 80% [8,9]. *BRCA1 *mutations also confer a medium to high risk of ovarian cancer [10]. Breast cancer is a highly heterogeneous malignancy, as demonstrated by gene expression microarray studies that proposed various molecular subtypes [11,12]. *BRCA1*-related breast cancers have been described to share similarities with basal epithelial (basal-like) and triple-negative phenotypes [13,14]. Basal-like breast cancers have an expression profile that is similar to that found in normal basally-positioned breast epithelial cells [11], and the majority of these are triple-negative. In other words, they do not express the genes estrogen receptor (ER), progesterone receptor (PR), and human epidermal growth factor receptor 2 (HER2/neu) [15]. Furthermore, when studied by immunohistochemistry, basal-like tumors are found to express one or more of cytokeratins 5, 14, and 17, c-KIT and epidermal growth factor receptor (EGFR) [16,17].

Recurrent gene fusions have been implicated in some forms of breast cancer, such as *ETV6-NTRK3 *in secretory breast ductal carcinoma [18]. The role of gene fusions in *BRCA1 *breast cancers, however, has not been well explored. One motivation to study them in these cancers is that BRCA1 is involved in many cellular processes as well as in repairing double-stranded DNA breaks (DSBs) mediated by homologous recombination (HR) [19]. HR is error-free and involves repairing DSBs by merging two broken ends based on sequence homology [20]. When BRCA1 is deficient in HR, evidence supporting increased activity of a second repair pathway, non-homologous end joining (NHEJ), has been shown [21]. While the role of BRCA1 in NHEJ is not well known, NHEJ is more vulnerable to errors as it involves repairing DSBs by incorporating or deleting nucleotides at the site of breakage to make the two broken ends compatible for ligation [22]. Errors resulting from NHEJ can lead to increased chromosomal aberrations, translocations, and unchecked DNA damage [23]. Furthermore, Stephens et al. [24] investigated genomic rearrangements in 24 breast cancer genomes with and without *BRCA1 *mutations and found genomic rearrangements to be significantly widespread. Hence, we hypothesized that deficiencies in BRCA1 would cause increased chromosomal instability in a tumor cell due to impaired DNA repair pathways and NHEJ dysfunction. The resulting chromosomal lesions may potentially lead to the creation of gene fusions that can be detected in the transcriptome.

We demonstrate the use of RNA-Seq to investigate the transcriptomes of *BRCA1*-mutated breast cancers for gene fusions. RNA-Seq is capable of producing single-end (SE) reads (i.e. reads sequenced from only one end of the cDNA fragment) or paired-end (PE) reads (i.e. both ends of the fragment are sequenced). We utilized both strategies for the discovery of gene fusions. Our analysis illustrates the feasibility of applying RNA-Seq to characterize gene fusions at the transcriptome level.

## Methods

### Sample collection and RNA-Seq preparation

We studied the transcriptomes of five *BRCA1*-mutated breast cancer cell lines (one of which is a matched lymphocyte cell line), three *BRCA1*-mutated primary tumors, two secretory breast ductal carcinoma primary tumors (SEC1 and SEC2) and one non-tumorigenic breast epithelial cell line (Table 1). To experimentally validate and detect for recurrence of our candidate gene fusions, we additionally obtained a cDNA panel of 57 breast cancers (19 *BRCA1*-mutated, 17 *BRCA2*-mutated, and 21 *BRCA1/2*-wild-type). Institutional Review Board-approved written informed consent forms were completed by all individuals whose tumor samples were used in this study.

**Table 1:** Summary of RNA-Seq samples

Sample name	Sample description	*BRCA1 *mutation	Sequencing strategy
HCC1937	Cell line	5266dupC	SE
SUM149PT	Cell line	2769delT	PE
SUM1315O2	Cell line	30_31delAG	PE
HCC3153	Cell line	815_824dup	PE
T92	Primary tumor	5266dupC	PE
T50	Primary tumor	4327C>T	PE
T160	Primary tumor	5521A>C*	PE
HCC2337	Lymphocyte cell line	5266dupC	PE
MCF10A	Non-tumorigenic breast epithelial cell line	-	PE
SEC1	Primary tumor	-	PE
SEC2	Primary tumor	-	PE

* See Lee *et al*. [58] for more details

Ten breast cancer samples (eight with known germline *BRCA1 *mutations and two secretory breast cancers) and one non-tumorigenic breast epithelial control was sequenced using the Illumina Genome Analyzer IIx. Only HCC1937 was sequenced using a single-end (SE) strategy, while the rest was sequenced using paired-end (PE). The cell line HCC2337 is a lymphocytic cell line derived from the same patient as HCC1937. Known *BRCA1 *mutations are based on RefSeq accession NM_008294.3.

For the samples subjected to RNA-Seq, we isolated 5 μg of total RNA from each sample and prepared them for high-throughput sequencing following the standard mRNA protocol by Illumina, unless specified otherwise. Briefly, mRNA from each sample was purified using Sera-mag magnetic oligo(dT) beads (Thermo Scientific) [for SUM149PT, Ribominus beads (Invitrogen) was applied to deplete ribosomal RNA species], fragmented at high temperatures into random fragments, and reverse-transcribed into cDNA using Superscript II (Invitrogen).

### Illumina Genome Analyzer sequencing

Each prepared cDNA library was subjected to high-throughput sequencing using the Illumina Genome Analyzer IIx platform, following the standard RNA sequencing protocol. Samples were sequenced at various sites and times and are described as follows: HCC1937 was sequenced by Illumina (50 bp SE). SUM149PT, SEC1 and SEC2 were sequenced at the Institute of Cancer Research (36 bp, 54 bp and 54 bp PE, respectively). SUM1315O2, HCC3153, HCC2337, T92, T50, T160 and MCF10A were sequenced at the McGill University and Genome Quebec Innovation Centre (76 bp PE per sample). All reads were processed using ELAND (AJ Cox, unpublished) to generate FASTQ files. Due to poor base quality at the 3' end of SUM1315O2 and HCC3153, reads in these samples were trimmed by 40 bp from the 3' end to produce 36 bp high quality PE reads. Additional read statistics are summarized in Additional file 1.

### RNA-Seq post-processing

Illumina FASTQ reads were first converted to Sanger FASTQ format using the ill2sanger script (patch by D Cittaro) from MAQ (http://maq.sourceforge.net/). Reads were then aligned to the human reference genome (hg18) using BWA [25]. Reads that span an exon-exon splice junction will not map to the reference since it will be separated by an intron. Thus, the remainder of the unmapped reads were aligned to a custom library of junction sequences based on known RefSeq annotation. For each splice junction and read length *x*, we joined *x*-5 bp (or the length of the exon if less than *x*-5) of sequence from the upstream exon with *x*-5 bp (or the length of the exon if less than *x*-5) of the downstream exon. This ensured that reads mapping to a splice junction overlapped to at least 5 bp of either end of the junction. The results were then processed using SAMTools [26] for downstream analysis. Visualization of the mapped reads was carried out using Integrated Genome Viewer (http://www.broadinstitute.org/igv).

### Identification of gene fusion transcripts from RNA-Seq

In this study, we implemented two strategies adapted from methods described by Maher et al. [3,4] to facilitate gene fusion discovery with SE reads and PE reads, respectively.

In the SE approach, gene fusions are identified by checking for reads which map across the exon-exon fusion junction between the two fused genes. To simplify the analysis, we reasoned that only the subset of reads which did not fully align to the reference genome or splice junctions would be interesting (unmapped reads), as they may harbour fusion junction spanning reads. All unmapped reads were realigned to RefSeq mRNA sequences using Blat [27]. Reads that partially aligned to a RefSeq sequence (20-70% identity) and did not fully map to anything else were retained. Ambiguous reads that were partially aligned to more than five genes were also discarded. Based on the position of the alignment, we further filtered for reads that partially aligned within 5 bp of an exon boundary using RefSeq annotation. This is because we expected that potential reads mapping across a gene fusion junction would be aligning at the exon boundaries. The resulting set of partially aligning reads was denoted as group A. Next, the unaligned portions of these reads were extracted and again subjected to alignment against RefSeq mRNA exon sequences using Vmatch [28], for effective alignment of shorter reads (<20 bp). Reads that fully mapped to an exon and localized within 5 bp of 5' end or 3' were retained in a similar fashion as before (denote as group B). Finally, we combined the results from group A with group B by finding reads that had a partial alignment in group A and a full alignment in group B, thus accounting for the entire read sequence and ultimately representing a gene fusion candidate read (GFCR). All the GFCRs were tallied and summarized. Fusion partners with greater than or equal to three unique GFCRs were considered a gene fusion candidate. Each candidate was manually examined to determine its potential biological significance and to check whether it could be a false positive. This included disqualifying reads that mapped to too many loci, which may represent a repetitive region, or gene fusion candidates which mapped to two homologs that were part of the same gene family.

For the PE approach, we searched for two features which may define a gene fusion. First, we limited our selection to reads that fully and uniquely mapped to the reference genome. Second, we reasoned that reads which map to different chromosomes may reflect an interchromosomal translocation and have the potential to form a gene fusion. Alternatively, reads that map to the same chromosome but at a larger than expected distance apart may represent an intrachromosomal translocation. However, this may be confounded by large introns situated between two exons that the reads map to. To account for this, we used an arbitrary minimum intrachomosomal mapping distance of 1 Mb. After identifying such read pairs, we realigned the reads to RefSeq mRNA sequences using Blat. Ensuring that each read in a pair mapped to a different gene, we summarized a list of candidate gene fusions in a similar fashion as for SE reads and generated a paired end fusion score (PEFS). The scoring scheme was defined as follows: +2 for each identified read pair, -1 for each duplicate read, and -0.5 for each mismatch in a read. We prioritized those that had a minimum of three read pairs that flanked a fusion junction and had a minimum PEFS score of 5. For further supporting evidence of each finding, we searched for additional reads that spanned across the fusion junction, analogous to the SE approach but restricted to a search space containing only the two partner genes. Here, we generated a library of all possible splice junction sequences between the 5' fusion partner and the 3' fusion partner, which includes the predicted fusion junction. All remaining unmapped reads were aligned to these junction sequences using Blat.

### Quantification of exon expression levels

Exon expression levels were quantified by first determining the number of reads that uniquely mapped to each exonic region using BEDTools [29]. The genomic coordinates for each exon were obtained from the UCSC Table Browser [30]. The number of reads was normalized and expressed as reads per kilobase of exon model per million mapped reads (RPKM) [31].

### Sanger sequencing

Primer pairs for PCR amplification and sequencing of each coding exon were generated using Primer3 [32]. Primers (see Additional file 2 for sequences) were designed to span the predicted exons forming the fusion breakpoint and to generate a maximum product size of 300 bp, which was considered optimal for amplification, purification, and sequencing. To minimize amplification of homologous genomic sequences, primer pairs were filtered using the UCSC In Silico PCR software, and only pairs yielding a single product were used. PCR reactions were performed on the cell lines' cDNA at least twice, using the following thermocycling parameters: 95°C × 15 min, (95°C × 20 s, 60°C × 20 s, 72°C × 20 s) for 30 cycles, 72°C × 10 min. PCR products were purified as recommended by the manufacturer (QIAGEN). Products were sequenced by conventional Sanger methods and compared to the reference sequence to validate the gene fusion. Sequence products were obtained from MUGQIC. Sequence chromatograms were aligned and analyzed with the Staden package and Mutation surveyor software version 3.24.

### RT-PCR

Total RNA (100 ng) was reverse transcribed into single-stranded cDNAs using SuperScript III reverse transcriptase (Invitrogen) and Oligo (dT)_12-18 _(Invitrogen) in 20 μl reaction at 50°C for 50 min, 85°C for 5 min, 37°C for 20 min. 2 μl of cDNA was used for a subsequent 20 μl PCR amplification. To detect fusion transcripts, we design the forward primer targeting the 5' partner gene and reverse primer targeting the 3' partner. Primer pairs (see Additional file 2 for sequences) for the coding exons of the fusion genes were generated using Primer3 [32]. GAPDH was amplified simultaneously. PCR was performed at an annealing temperature of 60°C for 26 cycles. PCR products were separated by gel electrophoresis in a 3% agarose gel (Metaphor, MRC) and visualized by ethidium bromide staining.

### Data access

Sequence data of the breast cancer cell lines (HCC1937, SUM149PT, SUM1315O2, HCC3153, HCC2337 and MCF10A) from this study has been deposited to the NCBI Sequence Read Archive (http://trace.ncbi.nlm.nih.gov/Traces/sra/sra.cgi) under accession no. SRA046769.

## Results

### Application of RNA-Seq to identify gene fusion transcripts

Initially in this study, we relied on using SE reads to identify gene fusions in the HCC1937 cell line. The remainder of this study was subsequently superseded by the PE approach, which relies on identifying read pairs that fully map to two different genes. To prioritize candidates for further review, we implemented a scoring scheme that assigned a paired end fusion score (PEFS) based on various characteristics of the read mapping. For example, a candidate with three mapped read pairs was awarded 2 points for each pair and thus had total PEFS of 6. However, a penalty of -1 was given for each read that had the same start coordinate as another supporting read. Such reads imply that they are duplicate reads and, although it is difficult to distinguish, may represent PCR duplicates originating from the amplification step during sequencing [33]. Hence, preference was given to candidate fusions that were supported by unique reads. Furthermore, by incorporating additional reads spanning the predicted fusion junction, the PE approach provides two unique pieces of evidence to support the discovery of gene fusion transcripts.

### Proof of concept

**Figure 1:**
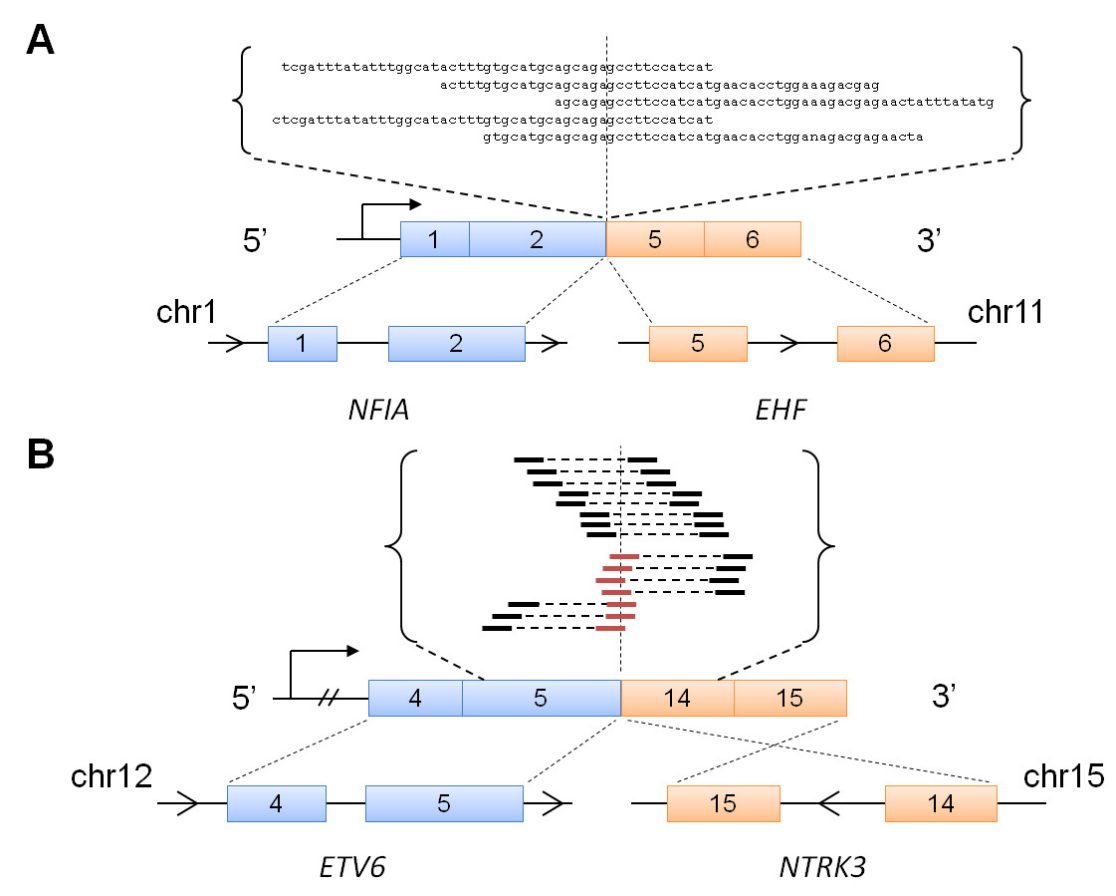
**RNA-Seq evidence of previously described gene fusions**. We first tested our SE approach on (A) the HCC1937 cell line that harbours the fusion *NFIA-EHF*. SE reads are shown to map across the exon-exon fusion junction between exon 2 of *NFIA *and exon 5 of *EHF*, as illustrated in the schematic. Next, we tested our PE approach on (B) two primary tumors that contain *ETV6-NTRK3*. Results from the sample SEC2 are shown. Paired reads (indicated by two solid lines joined by a dotted line) as well as single reads (red lines) are shown to map across the fusion junction between exon 5 of *ETV6 *and exon 14 of *NTRK3*.

To evaluate our implementation, we attempted to identify known gene fusions within our samples. We applied our SE approach on HCC1937. This cell line has been previously found to have a translocation involving exon 2 of *NFIA *on chromosome 1 joining with exon 5 of *EHF *on chromosome 11 using massively parallel DNA sequencing [24]. We were able to successfully confirm the expression of *NFIA-EHF *in the transcriptome of this cell line. Several 50 bp reads were mapped across the junction between exon 2 of *NFIA *and exon 5 of *EHF *(Figure 1A).

Next, we tested the PE approach on two primary tumors (SEC1 and SEC2) with secretory breast ductal carcinoma, a rare subtype of breast cancer. These samples were tested by other molecular methods to harbour the gene fusion *ETV6-NTRK3 *[34]. This fusion is caused by a balanced translocation, t(12;15)(p13;q25), joining exon 5 of *ETV6 *and exon 14 of *NTRK3 *and is recurrent in this cancer [18]. Furthermore, functional studies have established the fusion to have a causative role in the pathogenesis of this breast cancer subtype [35,36]. We successfully confirmed the gene fusion transcript in SEC2 by identifying read pairs that flanked the fusion junction as well as individual reads mapping across the fusion junction (Figure 1B). The reciprocal fusion, involving exon 13 of *NTRK3 *as the 5' partner gene and exon 6 of *ETV6 *as the 3' gene, was also identified in the same sample. On the other hand, we could not find the fusion in SEC1. Since no supporting PE reads were found, we checked to see if there were only individual reads that mapped across the *ETV6-NTRK3 *fusion junction. To test this, we mapped our reads to a custom-generated *ETV6-NTRK3 *junction sequence using Blat [27] and did not find any supporting reads. Subsequent inspection of starting sample from which SEC1 was prepared indicated that the tumor content was less than 50%. Hence, there may have been insufficient content of this gene fusion to be detected by RNA-Seq.

### Discovery of gene fusions

We analyzed the remaining samples for novel gene fusions. From the initial list of candidates, we found that many of the candidates with fewer than three supporting paired reads were due to duplicated reads, which may represent false positives. Samples T50 and T160, in particular, were sequenced at a higher depth than the other samples, yielding more sequencing data. A summary of the number of fusions found in each sample is shown in Table 2 In total, we found four candidates in samples with *BRCA1 *mutations that were supported by both misaligning PE reads and individual fusion junction-spanning reads. No fusions were identified in MCF10A. We manually inspected each of the four candidates and found that three of them formed in-frame transcripts, as described below and summarized in Table 3.

**Table 2:** Summary of candidate gene fusions identified by paired end RNA-Seq

Sample	*PEFS >*0	*PEFS *>= 5	*PEFS *>= 5 plus junction spanning reads
SUM149PT	1	1	1
SUM1315O2	20	2	0
HCC3153	8	1	1
T92	13	0	0
T50	1090	2	1
T160	437	4	1
HCC2337	14	2	0
MCF10A	10	0	0

In order to prioritize gene fusion candidates with the most read support, we selected candidates with a minimum *PEFS *of 5 for further investigation. A total of 12 gene fusion candidates with a *PEFS *>= 5 were identified based on discordantly aligned paired reads. Of these, four candidates (33%) were further found to have supporting reads spanning the exon-exon fusion junction.

**Table 3:** Candidate gene fusions supported by PE and junction spanning reads with PEFS >= 5

Sample	5' partner	3' partner	PEFS	In-frame?	Reference
HCC1937	*NFIA*	*EHF*	N/A	Y	[24]
SEC2	*ETV6*	*NTRK3*	5.0	Y	[18,34]
SUM149PT	*MTAP*	*PCDH7*	9.5	Y	-
HCC3153	*WWC1*	*ADRBK2*	5.0	Y	-
T50	*ADNP*	*C20orf132*	10.0	Y	-
T160	*CCDC126*	*HSP90AA1*	9.5	N	-

In addition to confirming two previously described gene fusions, we identified four novel gene fusions candidates that were selected based on our filtering criteria. Among these, three of the candidates were in-frame.

We identified a novel in-frame interchromosomal fusion transcript in SUM149PT involving exon 6 of *MTAP *on chromosome 9 joining with exon 3 of *PCDH7 *on chromosome 4. We found five read pairs flanking the fusion junction and two individual reads spanning the junction (Additional file 3A). The 5' gene, *MTAP*, encodes for a methylthioadenosine phosphorylase that has been frequently observed to be co-deleted with tumor suppressor gene encoding *p16 *in numerous cancers [37]. Interestingly, a deletion resulting in a fusion protein between *MTAP *and tumor suppressor gene encoding *p15^INK5B ^*was reported in a glioma xenograft as well as other malignant cell lines [38]. The 3' gene, *PCHD7*, encodes for an extracellular protocadherin protein involved in cell-cell recognition and adhesion. We were unable to amplify the *MTAP-PCDH7 *fusion junction using RT-PCR of cDNA in SUM149PT. One possible explanation for this failure could be due the low expression of both genes in this sample. We investigated this by examining their gene expression profiles in the RNA-Seq data, as will be discussed later.

**Figure 2:**
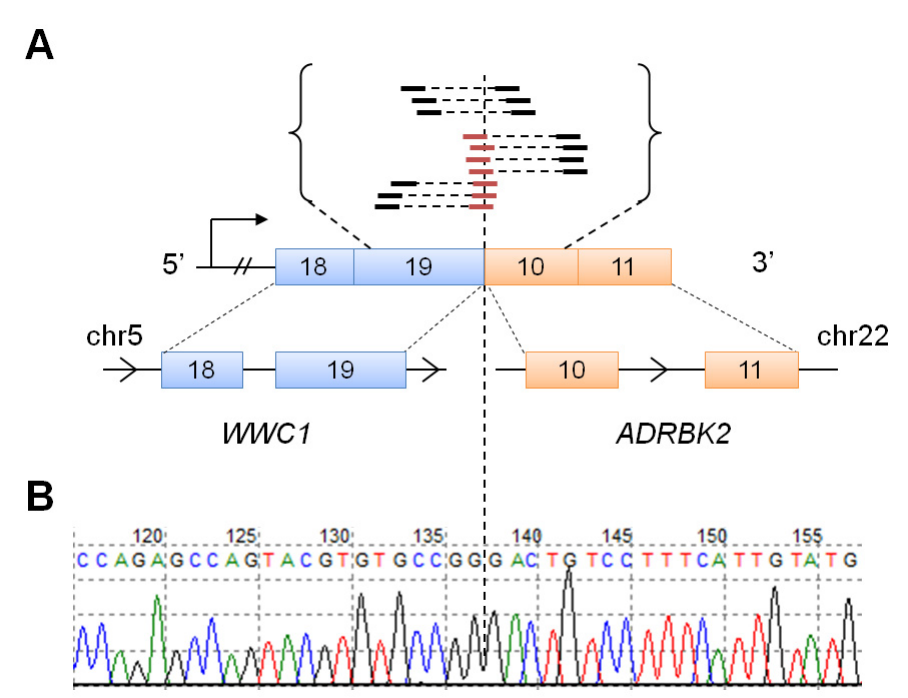
**RNA-Seq and Sanger sequencing of *WWC1-ADRBK2***. We identified an in-frame gene fusion transcript in HCC1315: (A) schematic of the predicted gene fusion illustrating RNA-Seq evidence that support the fusion between exon 19 of *WWC1 *and exon 10 of *ADRBK2*. Reads are indicated by black solid lines. Paired reads are indicated by the dotted line joining two reads. Reads that span across the fusion junction are highlighted by red solid lines; and (B) the fusion junction was verified using Sanger sequencing of cDNA.

A second in-frame interchromosomal fusion transcript was found in HCC3153 involving exon 19 of *WWC1 *on chromosome 5 joining with exon 10 of *ADRBK2 *on chromosome 22. We found three read pairs flanking the fusion junction and seven individual reads spanning the junction (Figure 2A). The 5' gene, *WWC1*, encodes for a protein KIBRA, a cytoplasmic phosphoprotein that regulates the Hippo/SWH signaling pathway and has been shown to be involved in tumor suppression [39]. The 3' gene, *ADRBK2*, encodes for a beta-adrenergic receptor kinase involved in the phosphorylation of G protein-coupled receptors. The ATP binding and kinase domains of this gene are conserved in the predicted fusion. To verify our finding, we performed PCR amplification of the *WWC1-ADRBK2 *cDNA at the fusion junction followed by Sanger sequencing (Figure 2B). Furthermore, in a separate experiment not described in this study, HCC3153 was subsequently re-sequenced at the Institute of Cancer Research using RNA-Seq on the Illumina Genome Analyzer. Our analysis of this data re-confirmed the expression of the predicted fusion transcript (data not shown). To determine for recurrence, we additionally screened a cDNA panel of 57 breast cancers and did not find the fusion in any of the cases.

**Figure 3:**
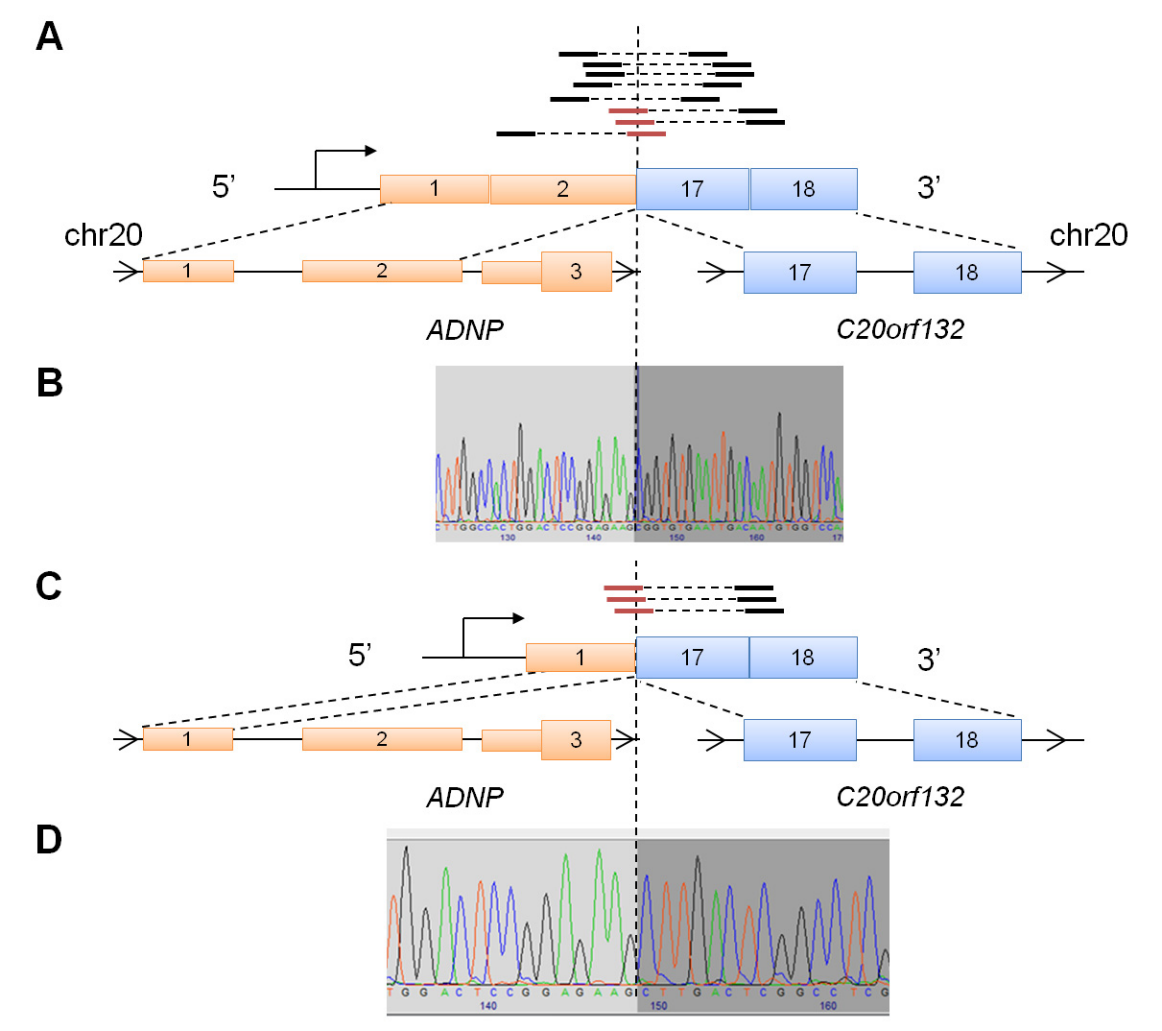
**RNA-Seq and Sanger sequencing of *ADNP-C20orf132***. We identified another in-frame gene fusion in a primary tumor that was present as two transcript isoforms: (A) schematic of the first predicted gene fusion isoform illustrating RNA-Seq evidence that support the fusion between exon 2 of *ADNP *and exon 17 of *C20orf132*; (B) Sanger sequencing of the fusion junction of the first isoform; (C) while searching for fusion junction-spanning reads, we subsequently identified a second isoform in which exon 1 of *ADNP *was fused with exon 17 of *C20orf132*; and (D) Sanger sequencing of the fusion junction of the second isoform. Reads are indicated by black solid lines. Paired reads are indicated by the dotted line joining two reads. Reads that span across the fusion junction are highlighted by red solid lines.

A third in-frame intrachromosomal fusion transcript was identified in a primary tumor T50 involving exon 2 of *ADNP *and exon 17 of *C20orf132*, both of which are located on chromosome 20 (Figure 3A). Interestingly, while identifying additional reads that mapped across the fusion junction, we found read evidence supporting an additional fusion isoform of *ADNP-C20orf132 *involving exon 1 of *ADNP *instead of exon 2 (Figure 3C). However, no PE reads were found to be discordantly mapped across this fusion isoform. Notably, exon 2 is known to be alternatively spliced in *ADNP*, resulting in a long and short wild-type transcript isoforms. Both of these two exons are located in the 5' UTR of *ADNP*, suggesting that this gene fusion may encode for a truncated version of *C20orf132*. *ADNP *encodes for an activity-independent neuroprotector homeobox protein that has been found to be involved in cell survival, due to its proximity to a region that is frequently amplified in cancer, 20q12-13.2 [40]. On the other hand, *C20orf132 *is an uncharacterized protein and no functional annotation was available. We validated both fusion transcript isoforms using Sanger sequencing (Figure 3B and 3D) and RT-PCR (Additional file 4) of cDNA from the primary tumor. Furthermore, we were not able to find evidence of this gene fusion expressed in the panel of 57 breast cancer samples discussed above, suggesting that it is not recurrent.

Lastly, by sequencing HCC1937 and HCC2337, a tumor cell line and matched lymphocyte cell line derived from the same patient, respectively, we sought to determine whether *NFIA-EHF *was a germline mutation or an acquired somatic mutation. Our analysis could not detect the expression of this gene fusion in HCC2337, suggesting that it represents the latter. As an additional observation, we confirmed via visual inspection of the mapped reads the presence of three mutations originally reported by Tomlinson et al. [41]: 1) a 5266dupC germline mutation in *BRCA1*; 2) a somatic deletion of *PTEN*; and 3) a somatic C->T substitution in *TP53 *(data not shown).

### Expression profiling of candidate gene fusions

We next studied the expression profiles of the candidate gene fusions based on our RNA-Seq data (Methods). The expression levels of every exon of each gene was quantified using a normalized metric described by Mortazavi et al. [31] called reads per kilobase of exon model per million mapped reads (RPKM). For each exon *e*, we examined the expression fold change (FC) ratio between the sample that the fusion gene is present in (denote as *e_S_*) and the average of our other fusion-negative samples (denote as *e_R_*). Formally, we define FC = RPKM(*e_S_*)/RPKM(*e_R_*).

**Figure 4:**
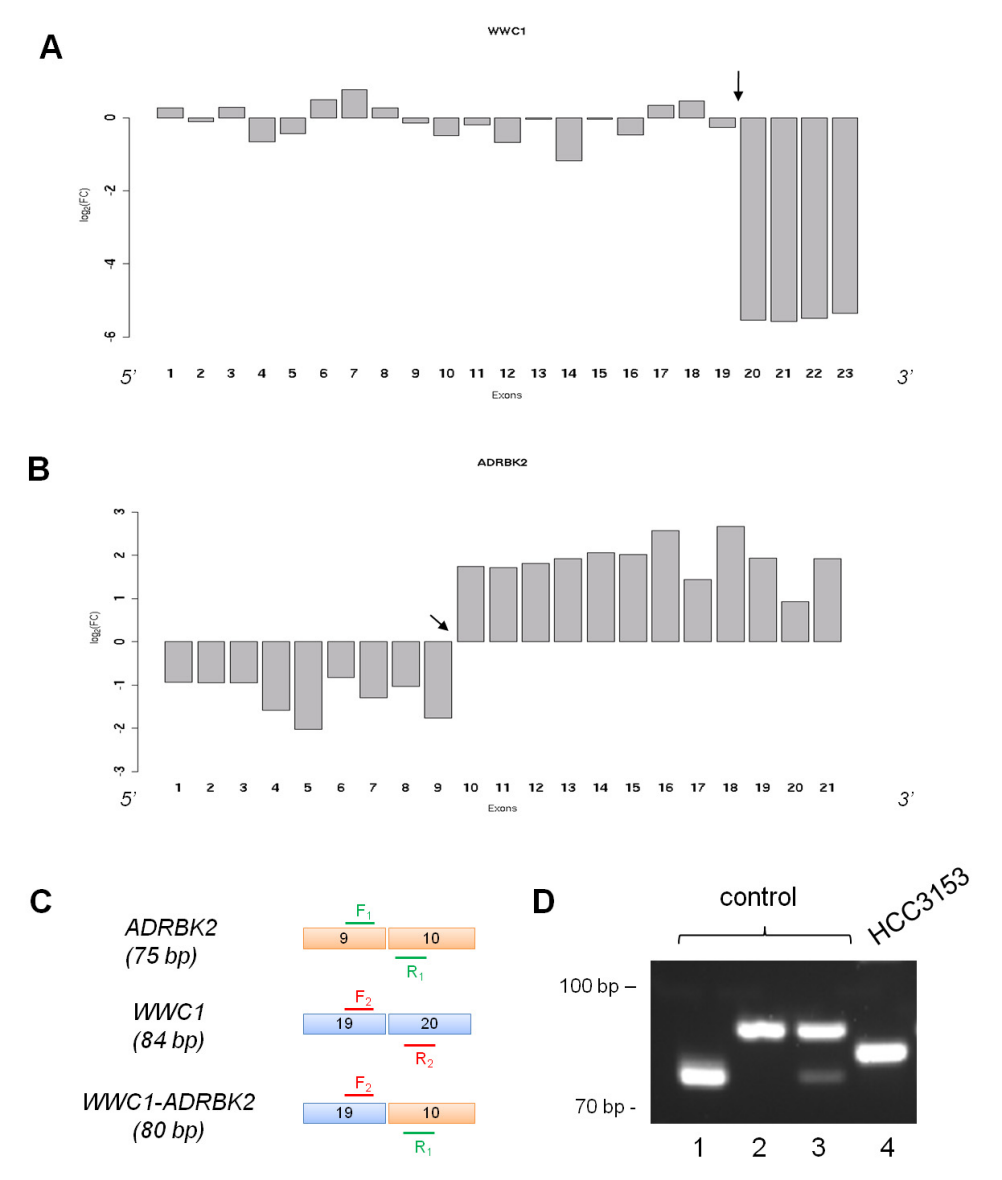
**Expression profile of WWC1 and ADRBK2**. We studied the gene expression profiles of *WWC1 *and *ADRBK2*: (A) Expression plot of *WWC1 *exons as measured by the log_2 _fold change (FC) between the RPKM values of each exon in HCC3153 versus the average of all other *WWC1-ADRBK2*-negative samples. The predicted gene fusion junction is marked by an arrow. It can be seen that exons downstream of exon 19 in *WWC1 *is underrepresented in HCC3153. (B) Similarly, the same can be observed in the exons upstream of exon 10 in *ADRBK2*. Moreover, the 3' end of the gene appears to be overexpressed. (C) Two sets of primers were designed to test for expression. The first set, F_1 _and R_1_, spanned exons 9 and 10 of *ADRBK2 *to test for wild-type expression. Similarly, the second set, F_2 _and R_2_, spanned exons 19 and 20 of *WWC1*. Both sets were also combined to test for expression of predicted *WWC1-ADRBK2 *fusion. The expected fragment sizes are shown in brackets. (D) Exon-exon RT-PCR results: a normal control was tested for the wild-type expression of *ADRBK2 *by F_1_/R_1 _(lane 1), the wild-type expression of *WWC1 *by F_2_/R_2 _(2), and with both sets together (3). In lane 4, both primer sets were applied on HCC3153 and confirmed expression of the expected fusion fragment but no wild-type expression.

Our analysis of *MTAP-PCHD7 *showed that both genes were not highly expressed in SUM149PT compared to the other samples. This was illustrated by plotting the log_2_-transformed FCs of all exons in each gene, as shown in Additional file 3B. Interestingly, for *WWC1-ADRBK2*, we observed a discordance in expression delineated at the predicted fusion breakpoint of both partner genes (Figure 4A-B). Furthermore, the retained 3' part of *ADRBK2 *showed increased expression over the other samples, which may be indicative of transcriptional activation driven by a foreign promoter or enhancer in *WWC1*. To experimentally confirm that the wild-type transcripts of these two genes were not expressed, we designed primers using RT-PCR (Figure 4C). As expected, only the fusion fragment was detected in HCC3153 (Figure 4D). Lastly, we repeated the exon expression analysis for *ADNP-C20orf132 *and similarly found discordant expression in *C20orf132*. Here, the exons at the 3' end of the *C20orf132 *(exons 17-24) were expressed higher than the 5' end (exons 1-16) (Additional file 4).

### Overlap with known copy number variation and chromosomal breakpoints

Gene fusions have been shown to be linked with genomic imbalances caused by copy number variation (CNV) [42]. While we do not have the necessary genomic data to interrogate CNVs within our samples for this study, we nonetheless explored whether the genes identified above overlapped with any known structural variation in the Database of Genomic Variants [43] and previously published array comparative genomic hybridization (aCGH) data on breast cancer cell lines [44]. In the former, at least one CNV was identified for all six genes examined (Additional file 5A-i and 5B). Among these, *ADRBK2 *had the most reported CNVs (n = 12) overlapping the 5' end of the gene. The majority of these CNVs, although rare, were centred approximately 300-500 kb upstream of *ADRBK2 *in a region that was enriched with segmental duplications. In the latter, the authors performed aCGH using 1 Mb resolution arrays to study CNVs in breast cancer cell lines including SUM149PT and HCC3153. To determine whether there were copy number changes near our genes, we examined the log_2 _fold changes of BAC clone(s) whose start position was within 1 Mb upstream and downstream of our genes (Additional file 5A-ii). For both *MTAP *and *PCDH7 *in SUM149PT, copy number losses were reported in BAC clones nearest these genes. In HCC3153, no BAC clones near *ADRBK2 *was found while copy number loss was reported near *WWC1*. However, more studies will be required to elucidate the relationship between these genes, their predicted rearrangement, and copy number within our specific cohort. Finally, to gain insight on the frequency of chromosomal breakpoints occurring near these genes, we consulted the Mitelman Database of Chromosome Aberrations and Gene Fusions in Cancer [45] to find previously reported breakpoints occurring near these genes (Additional file 5A-iii). The chromosomal region encompassing *ADRBK2 *was found to have the most number of cases with a reported breakpoint (n = 5193).

## Discussion

We have leveraged the power of massively parallel RNA sequencing to interrogate the transcriptomes of *BRCA1*-mutated breast cancer cell lines and tumors for putative gene fusions. In addition to identifying previously described gene fusions, we identified three novel in-frame fusions, *MTAP-PCDH7 *in SUM149PT, *WWC1-ADRBK2 *in HCC3153 and *ADNP-C20orf132 *in one primary tumor. Only the latter two were confirmed by RT-PCR and Sanger sequencing.

Gene fusions can adversely affect an organism by deregulating the normal expression and disrupting the function of genes. There are two main ways in which this occurs [46]. First, the active domain of one gene is joined with a regulatory enhancer or promoter of another gene, causing an upregulation of the active domain and leading to oncogenesis. Second, a hybrid or chimeric gene fusion is formed such that characteristics from both genes are active. Interestingly, for both *WWC1-ADRBK2 *and *ADNP-C20orf132*, we observed discordant expression delineated at the predicted breakpoint region of each gene. In both cases, expression was markedly higher in the 3' partner gene compared to samples that were negative for the gene fusion of interest. Hence, this suggests that they may represent examples of the first mechanism. Gene fusions are also known to be associated with CNVs [42]. While we observed some previously reported CNVs near the selected fusion genes, no conclusions could be drawn on their functional relationship.

None of the predicted gene fusions were found to be recurrent in any of the other samples that were sequenced. This raises the question of whether these fusions represent driver mutations that directly contribute to tumorigenesis or are passenger effects that have minor or no consequence. It is well understood that driver gene fusions are typically found to be recurrent, such as *BCR-ABL*, *ETV6-NTRK3*, and *TMPRSS2-ERG *in prostate cancer [47], and consequently they are ideal targets for therapeutic intervention. Since we were unable to detect any of our novel fusions in our screening of additional *BRCA1*-mutated, *BRCA2*-mutated or *BRCA1/2*-unrelated breast cancers, they may represent non-recurrent passenger mutations. However, more experimental studies will be required to elucidate their functional role. Moreover, many fusions that have been reported in literature have been found to be rare and have a low recurrence rate [48]. Hence, such low frequency gene fusions that are found in cancer may still be worth noting. If they can be observed in a patient, such private mutations can be potentially used as part of a personalized treatment program. For example, Leary et al. [49] recently demonstrated the ability to identify patient-specific genomic rearrangements as biomarkers in solid tumors using massively parallel sequencing. Indeed, our identification of *ADNP-C20orf132 *in a primary tumor represents one example of a private biomarker which may be used to track the status of the patient. Therefore, the use of sequencing-based approaches will be vital for advancing our understanding of tumors and to catalogue all known genetic abnormalities [50].

For this study, we focused our analysis on a collection of breast cancer samples with *BRCA1 *mutations. Tumors of this type may possess a distinctive, possibly unique, expression signature, despite their resemblance to basal-like and triple-negative breast cancers [51,52]. Hence, there has been great interest in elucidating the molecular mechanisms in *BRCA1 *cancers to identify potential biomarkers and drug targets. For example, genes that are more relied upon by tumor cells as a result of the loss of *BRCA1 *function can be targeted for inhibition and result in cell death. This synthetic lethal relationship has led to findings of potential drug targets that are sensitive to inhibition in *BRCA1 *tumors, such as mitogen-activated protein kinase [53] and poly (ADP-ribose) polymerase (PARP) [54]. In the case of the latter, a PARP inhibitor, olaparib (AstraZeneca) has already been developed and undergone successful clinical trials [54]. Knowledge of the role of gene fusions in *BRCA1 *breast cancers, however, is limited. In a study by Stephens et al. [24], gene fusions were identified in breast cancer genomes, but none of them were recurrent. We hypothesized that mutations in *BRCA1 *may increase the frequency of chromosomal aberrations due to defects in the DNA repair and NHEJ pathways. This in turn, could result in the expression of novel gene fusions that can be observed at the transcriptome level. As discussed above, our analysis of a limited number of samples did not reveal strong evidence of gene fusions as major contributors to the development of *BRCA1 *breast cancers. However, this does not discount other genomic instabilities and lesions that arise from *BRCA1 *mutations and are not detected by RNA-Seq. For example, studies have shown that BRCA1 has a role in centrosome function and the organization of chromosomes [55,56]. We expect future studies to involve analyzing a greater number of samples by massively parallel sequencing at the genomic and transcriptomic level, allowing for a more powerful and comprehensive interrogation of breast cancer.

We demonstrate the merits of using RNA-Seq to discover gene fusions. In particular, we note that our method to examine discordant expression between exons is related to a previously described approach to predict gene fusions using exon arrays [57]. However, while candidate fusion genes can be identified based on discordant exon expression, it can be difficult to determine which pair of genes is involved in the fusion. A sequencing-based approach can overcome this by additionally identifying reads that map across the exon-exon fusion junction. Initially, we explored using a strategy based on SE reads. Maher et al. [3] previously described an approach that leveraged both longer reads (> 250 bp) followed by short reads (35 bp) to find gene fusions. Since while initially working with HCC1937 we only had short 50 bp SE reads, a major challenge was to identify reads that partially aligned to two different genes. For example, a typical partial alignment may involve finding matching sequences less than 50% the length of the read (in this case, < 25 bp) that map to a gene. Given the large number of genes to be searched, it is likely that many of the shorter sequences will be matched in a non-specific manner. We mitigated this by filtering for matches that occur at or near the boundary of exons. Another approach was through the use of PE reads, which alleviated the reliance on finding junction spanning reads in the initial step [4]. Instead, we first systematically searched for paired reads that fully mapped to the genome but not at the expected distance or orientation, followed by searching for individual reads mapping across the predicted fusion junction. We also note two caveats to our approach. First, we identified candidate gene fusions based on RefSeq annotation for well-characterized content. As a result, some non-RefSeq genes may have been missed. Second, lowly expressed gene fusion transcripts are generally more difficult to detect if there is insufficient read coverage at the junction site and do not have supporting discordant PE reads. Thus, we focused on gene fusion candidates with adequate read coverage for further validation. In general, we found that the paired read property of PE reads allowed us to identify candidates with greater effectiveness than using only SE reads.

## Conclusions

The emergence of massively parallel RNA sequencing has enabled the direct profiling of transcriptomes and revolutionized transcriptomics research. With this technology, we found that the use PE reads is well-suited for the discovery of gene fusions in cancer via bioinformatics approaches. Our investigation of several *BRCA1*-mutated breast cancer cell lines and primary tumors revealed three previously uncharacterized non-recurrent gene fusions, two in cell lines and one in a primary tumor. While their functional role remains to be determined, no strong evidence was found to suggest that they are predominant in these cancers. However, their identification may serve as viable patient-specific biomarkers. Thus, the discovery and cataloguing of gene fusions will play an important role in the clinical treatment of solid tumours.

## Competing interests

The authors declare that they have no competing interests.

## Authors' contributions

KCHH implemented and performed the computational analysis for gene fusion discovery in RNA-Seq data. EL performed the initial alignment of reads to the reference genome and splice junctions library. CM, JH, CJL and AA contributed to early versions of sequence alignment pipelines. LL, LC and MBL designed and carried out experimental validation of the candidate gene fusions. LC performed further experimental testing on additional cohorts. RN, KF, IK and CJL carried out the massively parallel RNA sequencing at the London site. AVS provided the secretory breast carcinoma samples. MB provided the *BRCA1 *tumor samples. WDF provided the cell line samples. WDF also provided additional *BRCA1*/*BRCA2*-related and *BRCA1 *wild-type samples for validation. KCHH wrote the manuscript with contributions by EL, LC, JM and WDF. JM and WDF are co-principal investigators of this study. All authors have read and approved the final manuscript.

## Pre-publication history

The pre-publication history for this paper can be accessed here:

http://www.biomedcentral.com/1755-8794/4/75/prepub

## Supplementary Material

Additional file 1 **Read statistics of RNA-Seq samples**. A summary of read statistics of the RNA-Seq samples used in this study. Click here for file

Additional file 2 **Primer sequences for experimental validation**. This document contains the primer sequences used for experimental validation of candidate gene fusions (Sanger sequencing and RT-PCR). Click here for file

Additional file 3 **Schematic and expression profile of *MTAP-PCDH7 *gene fusion**. (A) Schematic illustrating paired-end reads that flank the fusion junction between exon 6 of *MTAP *and exon 3 of *PCDH7*. Reads are indicated by black solid lines. Paired reads are indicated by the dotted line joining two reads. Reads the span across the junction are highlighted by red solid lines; and (B) Expression plots of *MTAP *and *PCDH7 *as measured by the log_2 _FC between the RPKM values of each exon in SUM149PT versus the average of all other *MTAP-PCDH7*-negative samples. Click here for file

Additional file 4 **Expression profile of *ADNP-C20orf132 *gene fusion**. We extracted cDNA from primary tumor T50 and confirmed the presence of both isoforms of *ADNP-C20orf132 *by (A) RT-PCR. In both cases, we confirmed the presence of both isoforms (lanes 1 and 2) of this gene fusion. Lane 3 is a 50 bp ladder control. (B) Expression plots of *ADNP *and *C20orf132 *as measured by the log_2 _FC between the RPKM values of each exon in the T50 versus the average of all other *ADNP-C20orf132*-negative samples. Click here for file

Additional file 5 **Genomic features overlapping or near candidate fusion genes**. (A) We queried existing data to summarize known structural variation that overlaps or is within proximity of our candidate fusion genes; and (B) a list of overlapping CNVs reported in the Database of Genomic Variants. Click here for file
