# Supplementary material for: Identification of gene fusion transcripts by transcriptome sequencing in BRCA1-mutated breast cancers and cell lines
Source: BMC Med Genomics. 2011 Oct 27;4:75. doi: 10.1186/1755-8794-4-75 (PMC3227591; doi:10.1186/1755-8794-4-75)
Supplement: Additional file 1 — Read statistics of RNA-Seq samples. A summary of read statistics of the RNA-Seq samples used in this study. [file 1755-8794-4-75-S1.PDF]

# Additional File 1 – Read statistics of RNA-Seq samples

| Sample name | Number of lanes in flowcell used | Single-end (SE) or Paired-end (PE) | Read length | Total number of reads | Total number of mapped reads |
|---|---|---|---|---|---|
| HCC1937 | 3 | SE | 50 | 40,304,866 | 36,239,470 |
| SUM149PT | 2 | PE | 36 | 64,592,386 | 30,162,136 |
| SUM1315O2 | 4 | PE | 36* | 123,295,996 | 79,764,469 |
| HCC3153 | 4 | PE | 36* | 133,039,212 | 97,139,963 |
| T92 | 2 | PE | 76 | 252,388,932 | 192,074,702 |
| T50 | 4 | PE | 76 | 201,446,132 | 144,726,600 |
| T160 | 4 | PE | 76 | 204,554,346 | 193,913,756 |
| HCC2337 | 2 | PE | 76 | 117,586,560 | 73,738,904 |
| MCF10A | 2 | PE | 76 | 129,533,560 | 92,017,666 |
| SEC1 | 2 | PE | 54 | 88,642,118 | 62,397,294 |
| SEC2 | 2 | PE | 54 | 88,856,068 | 56,967,569 |

*Trimmed from 76 bp
