# Supplementary material for: Identification of gene fusion transcripts by transcriptome sequencing in BRCA1-mutated breast cancers and cell lines
Source: BMC Med Genomics. 2011 Oct 27;4:75. doi: 10.1186/1755-8794-4-75 (PMC3227591; doi:10.1186/1755-8794-4-75)
Supplement: Additional file 2 — Primer sequences for experimental validation. This document contains the primer sequences used for experimental validation of candidate gene fusions (Sanger sequencing and RT-PCR). [file 1755-8794-4-75-S2.PDF]

Additional File 2 – Primer sequences for experimental validation

A. *Sanger sequencing*

WWC1	Exon 19	TTAAAGGTGGACAAAGAGACCAA
ADRBK2	Exon 10	TTGTGAAAGGTGGTAGTGCAAAT
ADNP	Exon 1/5’ UTR 1	CCTCGAGGCCGAGTCAA
	Exon 2/5’ UTR 2	AGTGGTTATGCCCTGTACTACCA
C20orf132	Exon 17	TGTCTACAATGTATGGCAACAGG

B. *RT-PCR*

WWC1

Forward	CTCTAAGACCTTCTCCCCAGGAC
Reverse	TTTTGGACAGAGTGGAGCTGT

ADRBK2

Forward	GCCTTAAATGAAAGAATCATGTTG
Reverse	GGCATAGGTCATACATACAATGAAA

GAPDH

Forward	CTGCACCACCAACTGCTTAG
Reverse	GGACTGTGGTCATGAGTCCTTC
