# Supplementary material for: Identification of gene fusion transcripts by transcriptome sequencing in BRCA1-mutated breast cancers and cell lines
Source: BMC Med Genomics. 2011 Oct 27;4:75. doi: 10.1186/1755-8794-4-75 (PMC3227591; doi:10.1186/1755-8794-4-75)

Additional File 3 – Schematic and expression profile of *MTAP-PCDH7* gene fusion

A.

Illustration of paired-end reads that flank the breakpoint between exon 6 of *MTAP* and exon 3 of *PCDH7*. Reads are indicated by black solid lines. Paired reads are indicated by the dotted line joining two reads. Reads that span across the breakpoint are highlighted by red solid lines.

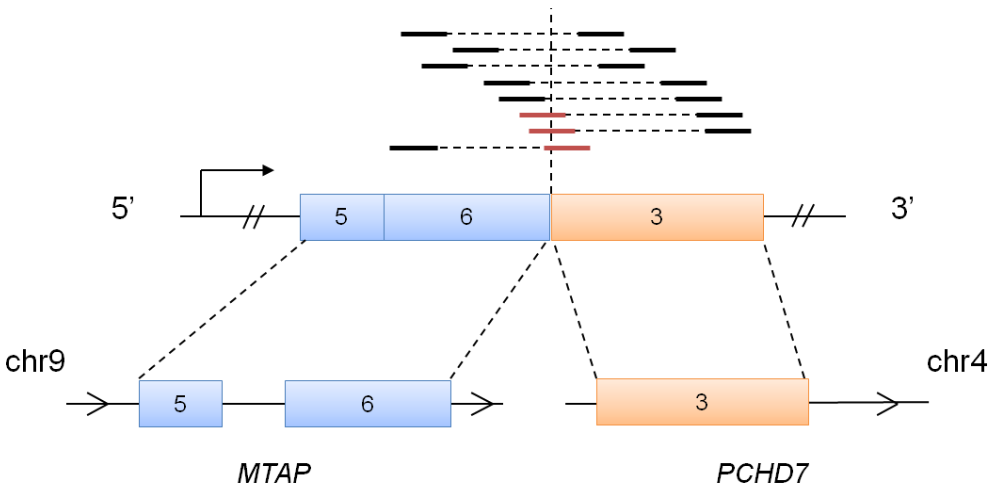

B.

Expression plots of *MTAP* and *PCDH7* as measured by the $\log_2$ FC between the RPKM values of each exon in SUM149PT versus the average of all other *MTAP-PCDH7*-negative samples.

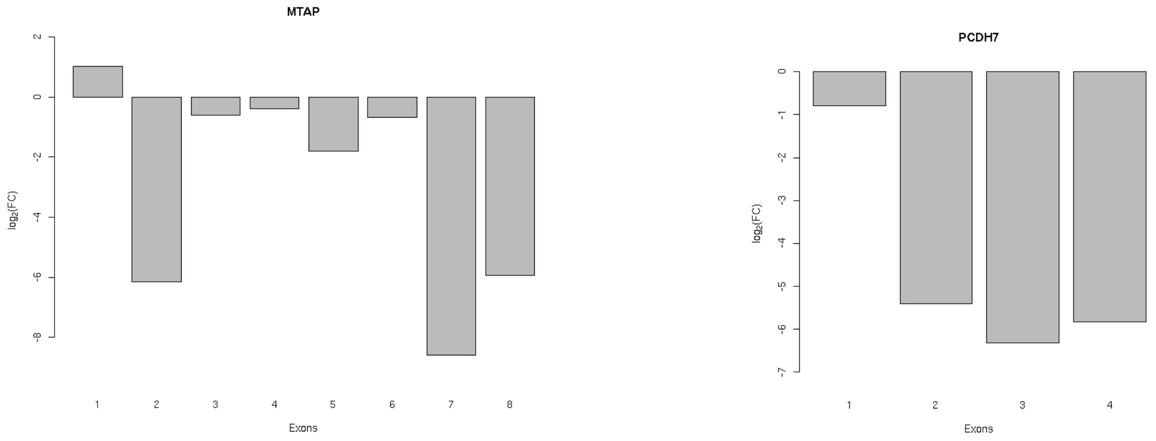

Supplement: Additional file 3 — Schematic and expression profile of MTAP-PCDH7 gene fusion. (A) Schematic illustrating paired-end reads that flank the fusion junction between exon 6 of MTAP and exon 3 of PCDH7. Reads are indicated by black solid lines. Paired reads are indicated by the dotted line joining two reads. Reads the span across the junction are highlighted by red solid lines; and (B) Expression plots of MTAP and PCDH7 as measured by the log2 FC between the RPKM values of each exon in SUM149PT versus the average of all other MTAP-PCDH7-negative samples. [file 1755-8794-4-75-S3.PDF]
