# Supplementary material for: Identification of gene fusion transcripts by transcriptome sequencing in BRCA1-mutated breast cancers and cell lines
Source: BMC Med Genomics. 2011 Oct 27;4:75. doi: 10.1186/1755-8794-4-75 (PMC3227591; doi:10.1186/1755-8794-4-75)

Additional File 4 – Expression profile of *ADNP-C20orf132* gene fusion

A.

Exon-exon RT-PCR of cDNA in primary tumor T50 shows expression of both *ADNP-C20orf132* transcript isoforms. Primers were designed to target the exons flanking the fusion boundary. Lane 1 illustrates the expression of a cDNA fragment (199 bp) featuring the fusion junction joining exon 1 of *ADNP* and exon 17 of *C20orf132*. Similarly, lane 2 illustrates the expression of a cDNA fragment (266 bp) featuring the fusion junction joining exon 2 of *ADNP* and exon 17 *C20orf132*. Lane 2 is a negative water control and lane 4 is a 50 bp ladder control.

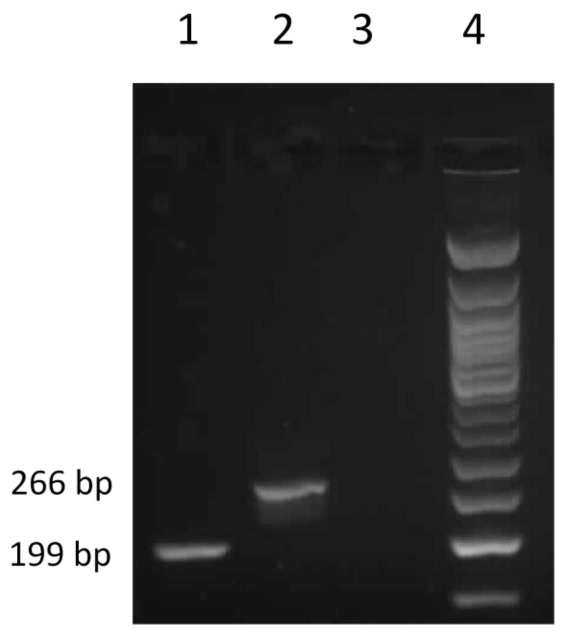

B.

Expression plots of *ADNP* and *C20orf132* as measured by the $\log_2$ FC between the RPKM values of each exon in primary tumor T50 versus the average of all other *ADNP-C20orf132* -negative samples. The ordering of the exons on both plots is reversed to match the reverse strand orientation of both genes (i.e. 3' – 5').

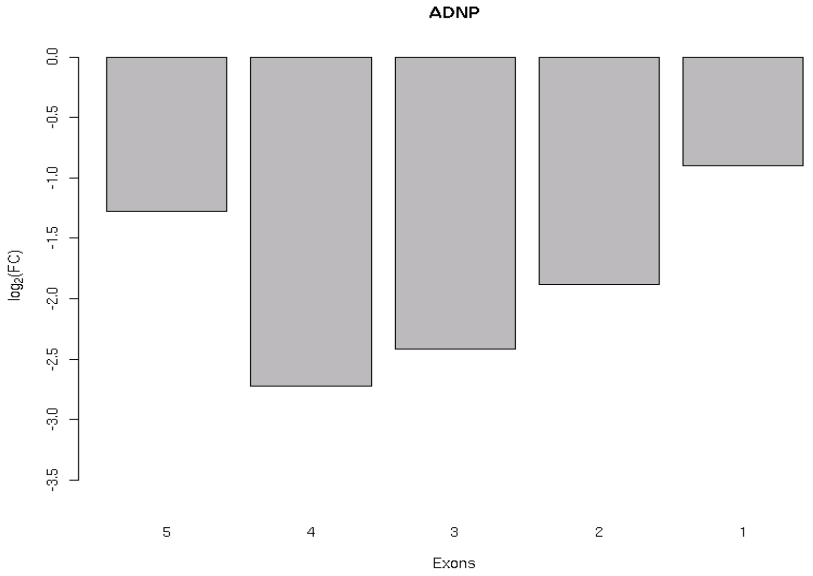

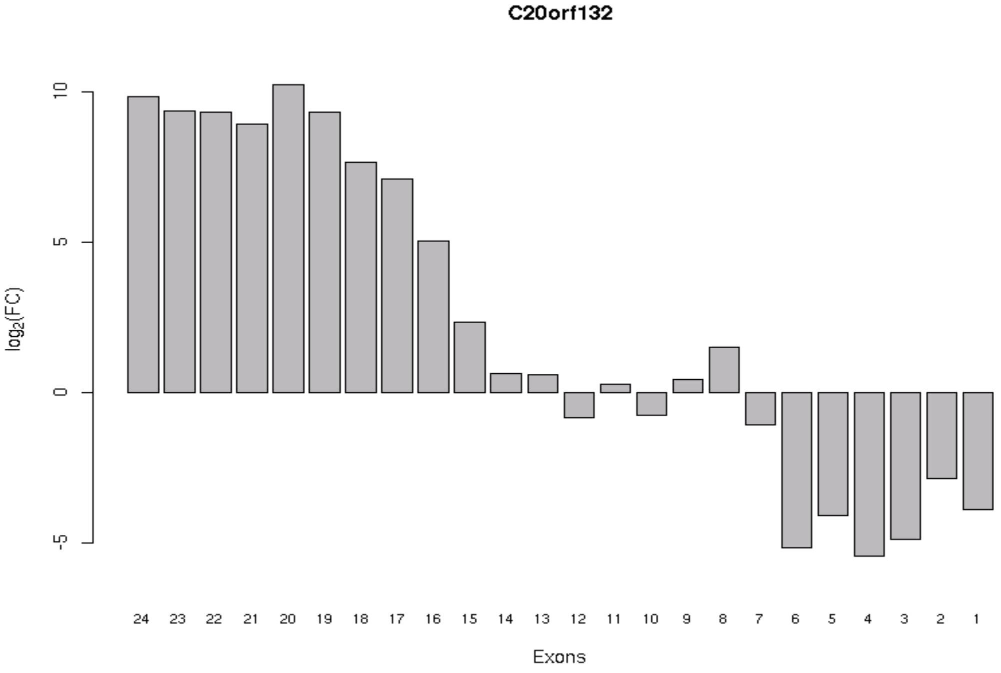

Supplement: Additional file 4 — Expression profile of ADNP-C20orf132 gene fusion. We extracted cDNA from primary tumor T50 and confirmed the presence of both isoforms of ADNP-C20orf132 by (A) RT-PCR. In both cases, we confirmed the presence of both isoforms (lanes 1 and 2) of this gene fusion. Lane 3 is a 50 bp ladder control. (B) Expression plots of ADNP and C20orf132 as measured by the log2 FC between the RPKM values of each exon in the T50 versus the average of all other ADNP-C20orf132-negative samples. [file 1755-8794-4-75-S4.PDF]
