# Supplementary material for: Identification of gene fusion transcripts by transcriptome sequencing in BRCA1-mutated breast cancers and cell lines
Source: BMC Med Genomics. 2011 Oct 27;4:75. doi: 10.1186/1755-8794-4-75 (PMC3227591; doi:10.1186/1755-8794-4-75)
Supplement: Additional file 5 — Genomic features overlapping or near candidate fusion genes. (A) We queried existing data to summarize known structural variation that overlaps or is within proximity of our candidate fusion genes; and (B) a list of overlapping CNVs reported in the Database of Genomic Variants. [file 1755-8794-4-75-S5.PDF]

Additional File 5 – CNVs from the Database of Genomic Variants associated with candidate fusion genes

A.

(i) We queried the Database of Genomic Variants [1] to gain insight into whether the predicted fusion genes overlapped with known CNVs previously reported in normal populations; (ii) using aCGH data of breast cancer cell lines from Neve et al. [2], we examined for CNVs in SUM149PT (for *MTAP* and *PCHD7*) and HCC3153 (for *WWC1* and *ADRBK2*). Only the BAC clone(s) with start positions within 1 Mb upstream and downstream the gene was considered. Based on the data, the average $\log_2$ FC was calculated. As defined in the original paper, copy number gains and losses were those with a log2 FC > 0.3 and < -0.3, respectively. There was insufficient data to determine CNVs in ADRBK2; (iii) the total number of chromosomal breakpoints reported in the Mitelman Database of Chromosome Aberrations and Gene Fusions in Cancer [3] that occur within or near the genes are shown. All cases and morphologies were considered.

**Gene symbol**	**Chromosomal locus**	**(i) Number of CNVs (gain/loss/both)**	**(ii) aCGH $\log_2$ fold changes from Neve et al. [2]**	**(iii) Number of reported breakpoints**
MTAP	9p21	0/1/0	-2.30	727
PCDH7	4p15	0/4/0	-0.35	214
WWC1	5q34-5q35.1	0/1/0	-0.41	422
ADRBK2	22q11-22q12.1	10/0/2	N/A	5193
ADNP	20q13.13	0/0/0	N/A	913
C20orf132	20q11.22	1/1/0	N/A	1175

B.

List of CNVs reported in the Database of Genomic Variants [1] that overlaps with our candidate fusion genes.

Gene	Gain/ Loss	Chromosomal Position	Frequency in study	Pubmed ID
MTAP	Loss	chr9:21,822,756-22,026,474	1/30	18304495
PCDH7	Loss	chr4:30424740-30447882	1/1	18451855
	Loss	chr4:30,594,659-30,596,133	1/1	19546169
	Loss	chr4:30,623,018-30,624,141	1/1; 16/30	20482838; 20364138
	Loss	chr4:30,679,037-30,685,504	1/36	16902084
WWC1	Loss	chr5:167,708,402-167,714,669	1/30	20364138
ADRBK2	Gain, loss	chr22:24,183,516-24,348,090	8/269	16826518
	Gain	chr22:23,991,725-24,324,013	1/485	18288195
	Gain	chr22:24,285,756-24,291,498	1/30	18304495
	Gain, loss	chr22:23,938,162-24,376,513	26/270	17122850
	Gain	chr22:23,980,648- 24,464,810	2/1190	17638019
	Gain	chr22:24,049,246-24,324,046	2/1190	17638019
	Gain	chr22:23,970,628-24,324,013	3/1064	19166990
	Gain	chr22:24,286,792-24,295,135	1/39	19166990
	Gain	chr22:24,183,516-24,348,090	2/47	15918152
	Gain	chr22:23,937,785-24,292,988	33/776	17911159
	Gain	chr22:23,988,546-24,480,646	1/776	17911159
	Gain	chr22:24,292,054-24,299,312	1/30	18304495
ADNP	--	--	--	--
C20orf132	Loss	chr20:35,231,723-35,233,420	1/36	16902084
	Gain	chr20:35,166,578-35,167,640	6/450	19812545

References:

1. Iafrate AJ, Feuk L, Rivera MN, Listewnik ML, Donahoe PK, Qi Y, Scherer SW, Lee C: **Detection of large-scale variation in the human genome.** *Nat Genet* 2004, **36:**949-951.
2. Neve RM, Chin K, Fridlyand J, Yeh J, Baehner FL, Fevr T, Clark L, Bayani N, Coppe JP, Tong F, et al: **A collection of breast cancer cell lines for the study of functionally distinct cancer subtypes.** *Cancer Cell* 2006, **10:**515-527.
3. **Mitelman Database of Chromosome Aberrations and Gene Fusions in Cancer** [http://cgap.nci.nih.gov/Chromosomes/Mitelman]
